# Associations between local descriptive norms for overweight/obesity and insufficient fruit intake, individual-level diet, and 10-year change in body mass index and glycosylated haemoglobin in an Australian cohort

**DOI:** 10.1186/s12966-018-0675-3

**Published:** 2018-05-18

**Authors:** Suzanne J. Carroll, Theo Niyonsenga, Neil T. Coffee, Anne W. Taylor, Mark Daniel

**Affiliations:** 1 Centre for Research and Action in Public Health, Health Research Institute, University of Canberra, Bruce, ACT Australia; 2 Spatial Epidemiology & Evaluation Research Group, School of Health Sciences and Centre for Population Health Research, University of South Australia, Adelaide, Australia; 3 Discipline of Medicine, The University of Adelaide, Adelaide, SA Australia; 4 Department of Medicine, The University of Melbourne, St Vincent’s Hospital, Melbourne, VIC Australia

**Keywords:** Descriptive norms, Cardiometabolic risk, Overweight and obesity, Dietary behaviour

## Abstract

**Background:**

Descriptive norms (what other people do) relate to individual-level dietary behaviour and health outcome including overweight and obesity. Descriptive norms vary across residential areas but the impact of spatial variation in norms on individual-level diet and health is poorly understood. This study assessed spatial associations between local descriptive norms for overweight/obesity and insufficient fruit intake (spatially-specific local prevalence), and individual-level dietary intakes (fruit, vegetable and sugary drinks) and 10-year change in body mass index (BMI) and glycosylated haemoglobin (HbA_1c_).

**Methods:**

HbA_1c_ and BMI were clinically measured three times over 10 years for a population-based adult cohort (*n* = 4056) in Adelaide, South Australia. Local descriptive norms for both overweight/obesity and insufficient fruit intake specific to each cohort participant were calculated as the prevalence of these factors, constructed from geocoded population surveillance data aggregated for 1600 m road-network buffers centred on cohort participants’ residential addresses. Latent growth models estimated the effect of local descriptive norms on dietary behaviours and change in HbA_1c_ and BMI, accounting for spatial clustering and covariates (individual-level age, sex, smoking status, employment and education, and area-level median household income).

**Results:**

Local descriptive overweight/obesity norms were associated with individual-level fruit intake (inversely) and sugary drink consumption (positively), and worsening HbA_1c_ and BMI. Spatially-specific local norms for insufficient fruit intake were associated with individual-level fruit intake (inversely) and sugary drink consumption (positively) and worsening HbA_1c_ but not change in BMI. Individual-level fruit and vegetable intakes were not associated with change in HbA_1c_ or BMI. Sugary drink consumption was also not associated with change in HbA_1c_ but rather with increasing BMI.

**Conclusion:**

Adverse local descriptive norms for overweight/obesity and insufficient fruit intake are associated with unhealthful dietary intakes and worsening HbA_1c_ and BMI. As such, spatial variation in lifestyle-related norms is an important consideration relevant to the design of population health interventions. Adverse local norms influence health behaviours and outcomes and stand to inhibit the effectiveness of traditional intervention efforts not spatially tailored to local population characteristics. Spatially targeted social de-normalisation strategies for regions with high levels of unhealthful norms may hold promise in concert with individual, environmental and policy intervention approaches.

## Background

Cardiovascular disease (CVD) and type 2 diabetes (cardiometabolic disorders) are preventable, yet combined these conditions represent approximately 40% of the health burden in Organisation for Economic Co-operation and Development (OECD) countries [1]. Similarly, the prevalence of overweight and obesity, risk factors for CVD and type 2 diabetes, continue to rise globally [2, 3]. Individual-level dietary choices are implicated in the development of overweight/obesity and cardiometabolic risk and disease, as well as other chronic diseases [4–8]. Diet-related diseases are among the leading health challenges of our time [4]. It is therefore essential to understand the drivers of individual dietary choices and how these relate to health outcomes.

Diets rich in fruits and vegetables, and low in sugar sweetened beverages are associated with better health outcomes [6, 9–12]. Such food consumption behaviours are influenced by a multitude of inter-related individual and environmental factors. At the individual-level these include age, sex, socioeconomic status (SES), attitudes to food and health, motivation and habit [13, 14]. In general, women, older adults, and individuals with higher incomes eat more fruits and vegetables [15–17].

Environmental influences on food consumption behaviours include physical and social environment factors. The physical environment includes accessibility and availability of various foods (e.g., healthful versus unhealthful food options) [13]. Literature reviews have documented reasonably consistent associations between accessibility to supermarkets and healthier diets and lower body weight, while greater accessibility to convenience stores and fast-food outlets is associated with poorer diets and higher body weight [18, 19].

A key influence on individual health behaviour and outcomes is the social environment which includes social pressures expressed as social norms [13]. A social norm is an expectation about behaviour that is shared by a group of people [20]. Social norms are collective constructs providing shared rules of conduct that can influence individual-level choices [21]. Such social norms can be conceptualised as two distinct, yet related, influences, injunctive and descriptive norms [22]. Injunctive norms represent what *ought* to be done, what is socially sanctioned while, descriptive norms are what most people actually do, what is *normal* [22]. A descriptive norm can therefore be considered the prevalence of a behaviour or trait [23, 24]. Though commonly conceived of as functioning within social networks, social norms also vary spatially (i.e., between and within areas). Previous research further defines social norms according to the source of influence, where “subjective” norms represent the influence of important others such as friends and family (e.g., social networks), and “local” norms represent the influence derived from people who are co-located regardless of any emotional connection [25–27]. Thus, a local descriptive norm can be considered as a *local* prevalence of a behaviour or trait [23, 28], an approach we have taken here.

Reviews of experimental studies concluded that social modelling and behavioural conformity shape food consumption amounts and choices (e.g., high energy versus low energy) [29–31]. Similarly, diet and dietary intentions have been linked in cross-sectional and longitudinal studies to the eating behaviours of social group members including family and friends [32, 33]. Social contagion studies have reported clustering and contagion (i.e., spread of a trait or behaviour over time) of alcohol consumption, smoking behaviour and obesity within social networks (i.e., subjective descriptive norms) [34–38].

Much of the research regarding the influence of social norms, injunctive or descriptive, has focused on social networks. The influence of spatially defined, local descriptive norms on health behaviour and outcomes has rarely been evaluated. One Dutch longitudinal study (13 years of follow up) reported a greater odds of an adult becoming overweight if they lived in a neighbourhood with a greater prevalence of overweight residents [39]. Our recent work found that local descriptive norms for overweight/obesity and insufficient fruit intake predicted worsening trajectories of HbA_1c_ [28]. However, a potential association between local descriptive norms and dietary intake of fruits, vegetables, or sugary drinks, or change in body mass index (BMI), was not assessed.

It is likely that local descriptive norms impact cardiometabolic outcomes including BMI and HbA_1c_ by influencing individual-level health behaviours such as dietary intake. Explicit testing of this mediating mechanism is needed to support biological plausibility of the link between local descriptive norms and health outcomes. Biological plausibility supports causal inference [40, 41]. Understanding the mechanisms linking local descriptive norms to health outcomes is necessary to inform the development of targeted intervention strategies. This study aimed to evaluate the direct effects of local descriptive norms for overweight/obesity and insufficient fruit intake on individual-level dietary intakes of fruits, vegetables and sugary drinks and 10-year change in HbA_1c_ and BMI. In addition, this study assessed whether individual-level dietary intake was a mediating mechanism linking local descriptive norms to change in health outcomes.

## Methods

This longitudinal observational study was part of the Place and Metabolic Syndrome (PAMS) Project which evaluated the role of residential environmental features in shaping individual-level cardiometabolic risk and received multiple ethics approvals (see Declarations). PAMS used data from the North West Adelaide Health Study (NWAHS), a population-based biomedical cohort of randomly selected adults (over 18 years of age) investigating the prevalence of chronic conditions and their associated risk factors [42]. Four thousand and fifty six of 8213 eligible adults participated in the NWAHS at baseline (49.4% of eligible sample). The NWAHS includes clinical data collected at three waves over 10 years: Wave 1 (2000–03, *n* = 4056), Wave 2 (2005–06, *n* = 3205, 79% of baseline sample) and Wave 3 (2008–10, *n* = 2487, 61.3% of baseline sample).

A geographic information system (GIS) was used to spatially join NWAHS data with data from the wholly separate South Australian Monitoring and Surveillance System (SAMSS) survey. The SAMSS is a population surveillance survey that monitors population trends in chronic diseases and their risk factors, geocoded at the street address [43, 44]. SAMSS data were used to construct local descriptive norms measures (environmental exposures).

### Study area

**Fig. 1:**
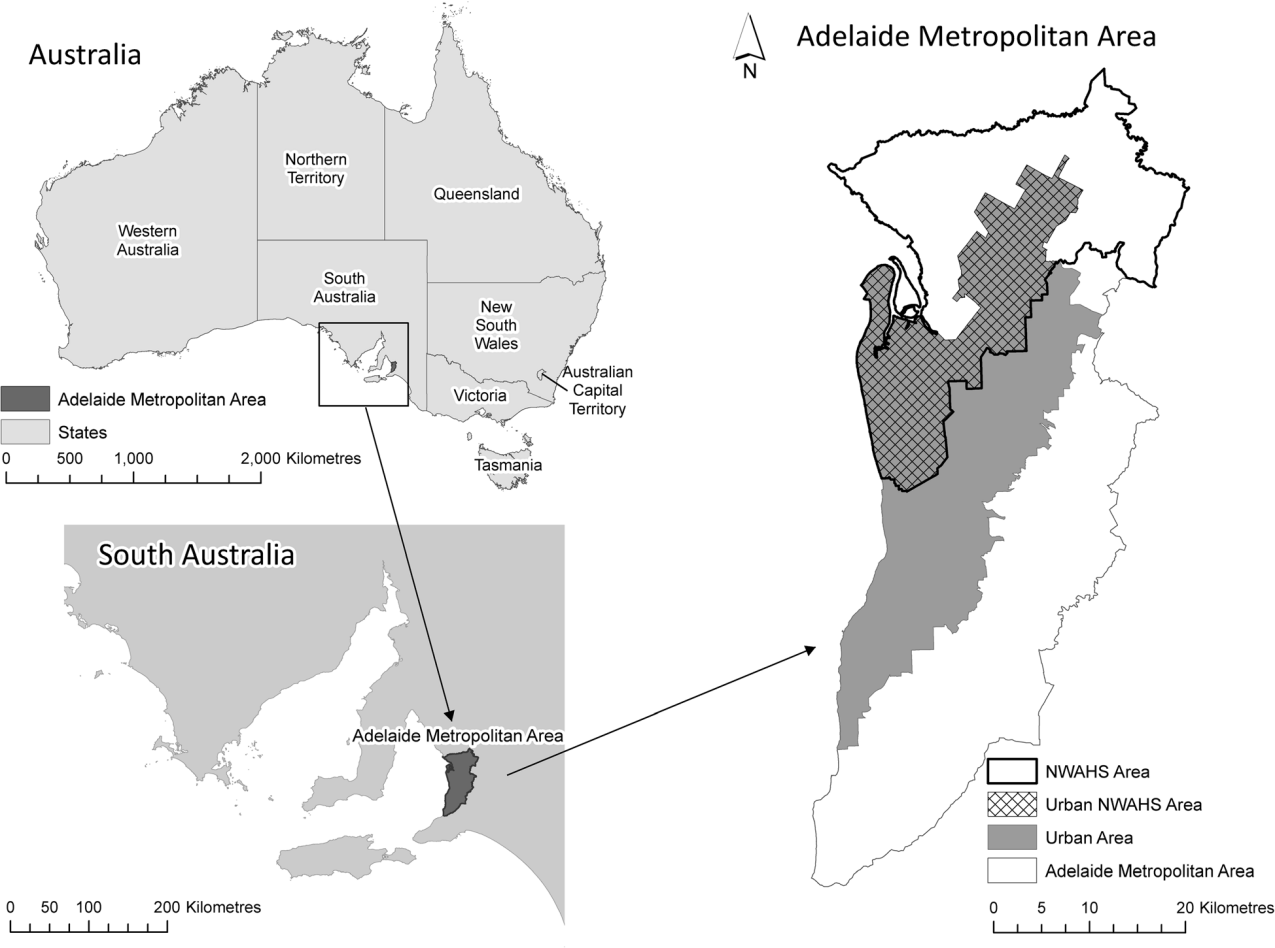
Study area - North West Adelaide region (urban only) (Reprinted from Social Science & Medicine, Vol. 166, Carroll, SJ, Paquet, C, Howard, N, Coffee, NT, Taylor, AW, Niyonsenga, T & Daniel, M, Local descriptive norms for overweight/obesity and physical inactivity, features of the built environment, and 10-year change in glycosylated haemoglobin in an Australian population-based biomedical cohort, pp. 233–243, 2016, with permission from Elsevier)

The NWAHS was conducted in the northern and western regions of metropolitan Adelaide (Fig. 1), the capital city of South Australia. In 2001 (baseline), these regions accounted for 38% of Adelaide’s 1.1 million population [45, 46].

Environmental associations with health behaviours and outcomes differ between urban and rural regions [47]. The study area was therefore limited to urban areas only, defined as Census Collection Districts (CDs) with a population density of > 200 persons per hectare and contiguous with other > 200 persons per hectare CDs [45].

### Cohort participants

Sociodemographic, behavioural and residential address (used to create a geo-reference point enabling spatial joining with other data) information were collected at each NWAHS wave using Computer-Assisted Telephone Interviews (CATIs) and self-reported paper questionnaires. Biomedical data, including fasting blood samples and anthropometric information, were collected during clinic visits at each wave.

Households within the NWAHS region (defined by postcode) were randomly selected from the Australian Electronic White Pages telephone directory and the adult (18 years or over) who most recently had their birthday asked to participate in the study. The baseline sample was not statistically different from the Adelaide metropolitan population [48] by sex, education or household income. However, older individuals (≥45 years) were over-represented and younger individuals (18–29 years) were under-represented. A multi-strategy approach was used to minimise cohort attrition, including the use of study promotional material, newsletters and birthday cards, and tracking based on the White Pages telephone directory and the State Electoral Roll [49]. Written informed consent was obtained prior to each data collection wave. Further information on data collection and cohort profile is available elsewhere [42, 49].

### Measures

#### Outcome measures

Outcome measures were HbA_1c_ concentration (%) and BMI calculated from height and weight measured during clinic visits at each wave. Height (without shoes) was measured using a wall-mounted stadiometer. Weight (without shoes, light clothing) was taken using standard digital scales. BMI was calculated as kilograms per metre squared (kg/m^2^). HbA_1c_ concentration, reflecting 2–3 month time-averaged blood glucose level [50], was assayed from fasting blood samples collected at each wave [42].

### Local descriptive norms

Local descriptive norms for overweight/obesity and insufficient fruit intake were defined within road-network buffers constructed by radiating 1600 m (1 mile) along the road-network in all possible directions from each participant’s residential address. The 1600 m distance represents the distance covered by an average adult walking at a comfortable pace (5 km/hour) for approximately 20 min [51] and has previously been used in studies assessing the impact of the local food environment on health outcomes (e.g., [52–54]).

Concordance between the 1600 m road-network buffers and geocoded SAMSS participant addresses (for adults 18 years and over) was established using a GIS. Individual-level SAMSS data were extracted according to this concordance, then spatially aggregated to construct buffer-specific prevalence rates for overweight/obesity (BMI ≥ 25 kg/m^2^) and insufficient fruit intake (< 2 servings per day), per standard health recommendations [55, 56]. As appropriate weightings for standardisation were not available for the buffers used, prevalence rates were unstandardised in keeping with the precedent of Blok and colleagues [39]. The use of other weightings such as for the Adelaide metropolitan region could artificially reduce or inflate spatial variation. Processing of individual-level data was performed by the data custodians to protect the confidentiality of SAMSS participants. More information on the SAMSS is available elsewhere [43, 44].

Geocoded SAMSS data were not available for years prior to 2006. Hence, local descriptive norms are not contemporaneous with the NWAHS baseline but instead represent normative exposures from Wave 2 to 3. To maximise SAMSS participant numbers for NWAHS participant buffers, data were pooled across 2006–2010. Aggregated norms data for buffers with less than 50 SAMSS participants or less than five participants per measurement category were not released by data custodians. This protects the confidentiality of SAMSS participants and provides more reliable local descriptive norms estimates. Detail on the construction of local descriptive norms measures is available elsewhere [21, 28]. Local descriptive norms measures were standardised (i.e., z-scores with a mean of zero) prior to analyses.

### Individual-level dietary intakes

Individual-level dietary measures were self-reported fruit, vegetable and sugary drink intakes. Such dietary intake information was collected in 2007 using an additional CATI subsequent to the main Wave 2 CATI. The method of collection for dietary information was questions from the Australian National Health Survey [57]. Information included self-reported usual daily servings of fruits and vegetables, and weekly frequency of sugary drink consumption. Fruit and vegetable questions asked participants to report the usual number of serves of fruits and vegetables they consumed per day (with examples of what constitutes a serve). Questions about the frequency of soft drink, cordial or sports drinks had a range of reporting options from “never”, “rarely”, or number of times per year through to number of times per day. For our purposes, sugary drink consumption responses were recoded for expression as frequency per week with “rarely” and “never” coded as zero consumption per week.

Due to their inclusion in statistical models as both predictors and outcomes, fruit and vegetable intake measures (usual serves per day) were log transformed to improve their distributional characteristics. Log transformation of sugary drink consumption (weekly frequency) was considered but rejected due to the zero-inflated distribution. Thus, a two-category version of the measure was constructed, defined as no sugary drink intake versus sugary drink intake. Other categorisations were considered (e.g., three categories) but given the complexity of the SEM models with sugary drinks analysed as a mediating variable (i.e., involving use as both an outcome and a predictor), ordinal expression was not possible. Use of the three-category version (as two dummy coded variables) within models resulted in large increases in the AIC and the BIC indicating that the two-category version had better model fit.

### Covariates

Individual and area-level covariates included in statistical models were individual-level age, sex, employment status (full-time, part-time, or not in the work force), level of education (university graduate or not), marital status (married/de facto, or single) and smoking status (current smoker or non-smoker), and area-level income (median household income).

Individual-level covariates were selected based on previous research regarding dietary behaviour and cardiometabolic risk, and analyses to identify factors predicting loss to follow-up (logistic regression was used to determine variables associated with attrition, defined as nonattendance for clinical assessment) [21]. Incomplete clinic attendance (missing HbA_1c_ and BMI information at follow ups) was predicted by male sex, young age, low household income, not being in the work-force, being a smoker, and not being married (or de facto). These measures were therefore included in statistical models to satisfy the analytic criterion of *missing at random* [58].

Area-level SES was operationalised as median weekly household income, commonly used in research assessing associations between area-level SES and health [59]. To avoid multicollinearity, only one area-level SES measure was included in the models. Data were extracted from the 2006 Australian Population and Housing Census [60] at the smallest available unit, the Census District (CD, average of 220 dwellings [61]), and aggregated using the weighted average of values from CDs intersected by the NWAHS participant buffers. Further information on this method is available elsewhere [21, 28].

### Analyses

**Fig. 2:**
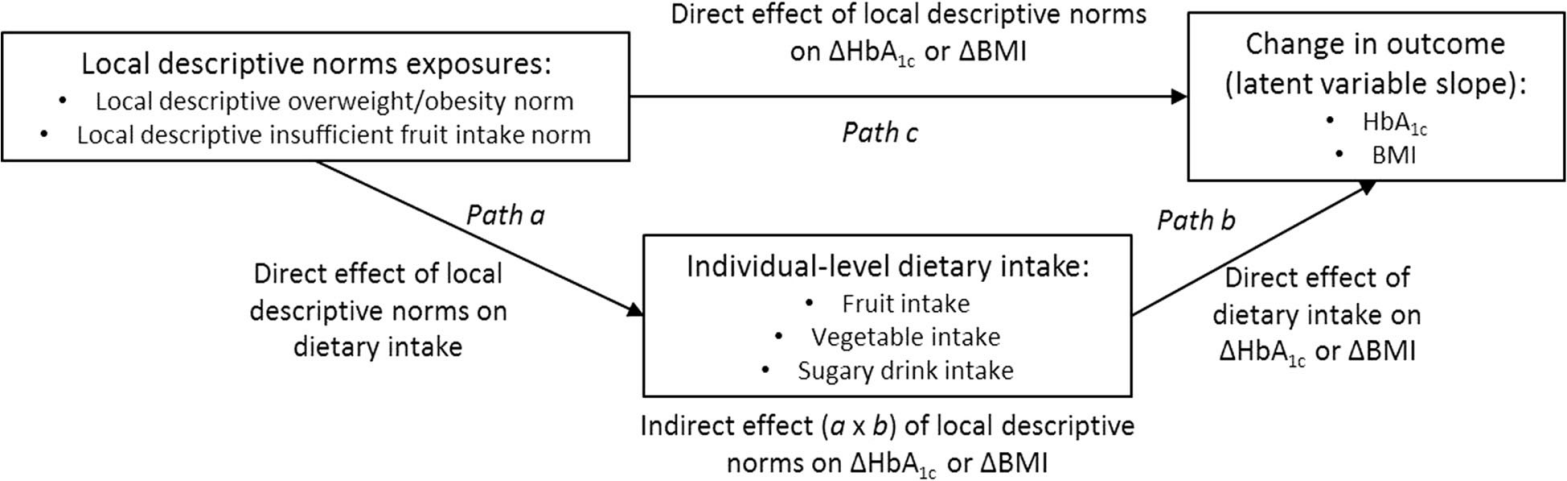
Path diagram of effects tested using structural equation modelling

Figure 2 illustrates the direct and indirect effects being tested. These effects were estimated using latent growth models in Mplus (version 7.4, Muthen & Muthen) with a structural equation modelling (SEM) approach and a Monte Carlo integration estimation-based process [62, 63]. Full information maximum likelihood (FIML) was used to estimate parameter estimates with standard errors (computed using a sandwich approach) robust to non-normality and non-independence of observations. Use of FIML allowed the inclusion of cases with missing dietary information [64, 65]. The SEM approach enables the simultaneous estimation of direct and indirect effects within one model as opposed to the use of multiple regression models [62, 66, 67].

Trajectories of outcomes, HbA_1c_ and BMI, were modelled as latent growth factors with random effects for participant variation in baseline values (intercept), and participant-specific rate of changes in outcomes (slope) over time. Each model estimated the influence of a single local descriptive norm on one dietary intake (e.g., fruit intake) and the rate of change in one outcome variable (latent growth factor slope for HbA_1c_ or BMI), and the influence of dietary intake on rate of change in the outcome variable. Models accounted for spatial clustering within suburbs and are reported both unadjusted and adjusted for individual-level covariates and area-level income predicting outcome latent growth factor intercept and slope. Indirect effects were calculated as the product of coefficients for *path a* and *path b* using the model constraint estimation approach [62]. Statistical significance was set at alpha = 0.05.

Separate sets of analyses assessed relationships with the two outcome measures, HbA_1c_ and BMI. Each analysis sample was restricted to individuals who did not have CVD or diabetes type 2 at baseline (HbA_1c_ sample), or who were not obese (obese being defined as BMI > 30) at baseline (BMI outcome sample). These restrictions were applied to reduce the influence of a potential ceiling affect as, on average, the NWAHS cohort had worsening health trajectories. This aligns with health expectations based on associations between aging and normative, age-related increases in HbA_1c_ and BMI.

## Results

Sample loss due to inclusion criteria is presented in Table 1. From the 2797 NWAHS participants meeting the initial inclusion criteria, four sub-samples (highlighted in bold in Table 1) were constructed based on outcome measure and availability of local-area descriptive norms information.

**Table 1:** Inclusion criteria and derivation of the three analytic samples

Criteria	n		Reason for reduced numbers
NWAHS sample (W1)	4056		–
Geocoded (W1)	4041		15 participants with invalid residential addresses
Residing in urban area (W1)	3887		154 participant addresses outside the urban area
Participated in Wave 2	3362		525 participants did not participate in Wave 2
Did not move (W1 to W2)	2797		565 participants moved between Waves 1 and 2
	*HbA*_*1c*_	*BMI*	*Of participants meeting previous criteria (n = 2797):*
CVD/diabetes free at Wave 1	2325	–	472 participants had CVD or Type 2 diabetes at Wave 1
Not obese at Wave 1 (BMI < 30)	–	1982	815 participants were obese at Wave 1
Covariate data (W1)	2261	1926	64 (HbA_1c_ sample) and 56 (BMI sample) participants lacked covariate data at Wave 1
LDN: Overweight/obesity	1908	1630	353 (HbA_1c_ sample) and 296 (BMI sample) participants lacked overweight/obesity norm data
LDN: Insufficient fruit intake	1966	1673	295 (HbA_1c_ sample) and 253 (BMI sample) participants lacked local insufficient fruit intake norm data

Abbreviations: *BMI* body mass index, *CVD* cardiovascular disease, *HbA*_*1c*_ glycosylated haemoglobin, *LDN* local descriptive norms, *NWAHS* North West Adelaide Health Study, W1, Wave 1; W2, Wave 2

Characteristics of the four analytic samples and the features of their environments are provided in Table 2. There were no notable differences between the two HbA_1c_ analytic samples, or between the two BMI analytic samples.

**Table 2:** Sample characteristics and features of areas for each of the four analytic samples

	ΔHbA_1c_ analytic samples		ΔBMI analytic samples	
Measure	LDN: Overweight/obesity *n* = 1908	LDN: Insufficient fruit intake *n* = 1966	LDN: Overweight/obesity *n* = 1630	LDN: Insufficient fruit intake *n* = 1673
Individual-level characteristics	Mean (SD)	Mean (SD)	Mean (SD)	Mean (SD)
Length of follow-up (years)	7.85 (1.05)	7.85 (1.05)	7.90 (1.03)	7.89 (1.03)
Age (years)	49.9 (15.2)	49.9 (15.2)	51.6 (16.3)	51.6 (16.2)
Sex (female) n (%)	1052 (55.1%)	1085 (55.2%)	830 (50.9%)	853 (51.0%)
Current smoker n (%)	335 (17.6%)	345 (17.6%)	288 (17.7%)	294 (17.6%)
Education (university graduate) n (%)	250 (13.1%)	254 (12.9%)	211 (12.9%)	213 (12.7%)
Marital status (married) n (%)	1227 (64.3%)	1260 (64.1%)	1031 (63.3%)	1060 (63.4%)
Not employed n (%)	816 (42.8%)	844 (42.9%)	748 (45.9%)	768 (45.9%)
Fruit intake (daily count of servings)	(*n* = 1587) 1.5 (1.0)	(*n* = 1638) 1.5 (1.0)	(*n* = 1337) 1.5 (1.0)	(*n* = 1375) 1.5 (1.0)
Vegetable intake (daily count of servings)	(*n* = 1583) 2.5 (1.4)	(*n* = 1633) 2.5 (1.4)	(*n* = 1332) 2.5 (1.4)	(*n* = 1370) 2.5 (1.4)
Drinks sugary drinks n (%)	(*n* = 1588) 923 (58.1%)	(*n* = 1639) 952 (58.1%)	(*n* = 1337) 730 (54.6%)	(*n* = 1375) 751 (54.6%)
HbA_1c_	(*n* = 1904) 5.43 (0.45)	(*n* = 1962) 5.43 (0.45)	(*n* = 1610) 5.52 (0.66)	(*n* = 1653) 5.52 (0.67)
BMI (kg/m^2^)	27.57 (5.20)	27.59 (5.20)	25.27 (2.86)	25.28 (2.86)
Environmental features	Mean (SD)	Mean (SD)	Mean (SD)	Mean (SD)
1600 m buffer area (km^2^)	4.71 (2.40)	4.72 (2.41)	4.78 (2.41)	4.78 (2.42)
LDN: Overweight/obesity	62.85 (6.18)	–	62.65 (6.15)	–
n _(SAMSS participants)_ per buffer	95.6 (31.5)	–	97.2 (31.7)	–
LDN: Insufficient fruit intake	–	53.79 (6.57)	–	53.36 (6.63)
n _(SAMSS participants)_ per buffer	–	100.3 (33.6)	–	102.0 (33.6)
Area-level median household income (A$/week)	838.45 (131.64)	837.13 (134.52)	839.06 (130.29)	838.15 (132.57)

Abbreviations: *BMI* body mass index, *HbA*_*1c*_ glycosylated haemoglobin, *LDN* local descriptive norms, *SD* standard deviation

Intraclass correlations (ICCs) were calculated from covariance parameter estimates obtained from a multilevel (three-level) empty model (i.e., with no predictors) performed in SAS (version 9.4, SAS Institute Inc., Cary, North Carolina) [68]. These ICCs describe the degree of similarity of HbA_1c_ concentrations or BMI for repeat measures within participants and for clustering of participants within suburbs. Estimates indicated moderate correlation at the individual-level (repeated HbA_1c_ measures over time: ICC = 0.57; repeated BMI measures over time: ICC 0.80) and relatively low to very low correlation at the suburb level (HbA_1c_ ICC = 0.01; BMI ICC = 0.002), consistent with previous reports [69].

Results of the fitted analytic models are presented in Tables 3 and 4. Estimation of the latent growth factors for HbA_1c_ (with no predictors) indicated average baseline HbA_1c_ of 5.42% (95% confidence interval [CI] 5.39 to 5.44, *p* < 0.0001), and an annual increase (worsening) of 0.04 percentage points (95% CI 0.029 to 0.040, *p* < 0.0001) (results not shown). Similarly, average baseline BMI was 25.26 kg/m^2^ (95% CI 25.12 to 25.39 kg/m^2^, *p* < 0.0001) with an annual increase of 0.12 kg/m^2^ (95% CI 0.10 to 0.14, *p* < 0.0001).

**Table 3:** Results of structural equation models with change in HbA_1c_ as outcome

**LDN: Overweight/obesity ** ***N*** ** = 1908**	**Unadjusted models**			**Adjusted models** ^a^		
Fruit serves (log transformed)	Estimate	95% CI	*P* value	Estimate	95% CI	*P* value
ΔHbA_1c_ on LDN (overweight/obesity)	**0.008**	**0.003 to 0.012**	**0.001**	**0.009**	**0.004 to 0.013**	**0.000**
ΔHbA_1c_ on fruit intake_(log)_	−0.004	− 0.010 to 0.002	0.241	− 0.004	− 0.012 to 0.003	0.259
Fruit intake_(log)_ on LDN (overweight/obesity)	**− 0.025**	**− 0.047 to − 0.002**	**0.030**	**− 0.025**	**− 0.047 to − 0.002**	**0.030**
Indirect effect *100	0.009	− 0.009 to 0.027	0.316	0.009	− 0.010 to 0.028	0.347
Total effect *100	**0.795**	**0.352 to 1.238**	**0.000**	**0.881**	**0.423 to 1.340**	**0.000**
Model fit	**–**	AIC 6485.203	BIC_adj_ 6518.479	–	AIC 6063.327	BIC_adj_ 6129.877
Vegetable serves (log transformed)						
ΔHbA_1c_ on LDN (overweight/obesity)	**0.008**	**0.003 to 0.012**	**0.001**	**0.009**	**0.004 to 0.013**	**0.000**
ΔHbA_1c_ on vegetable intake_(log)_	−0.003	− 0.010 to 0.005	0.456	− 0.001	− 0.009 to 0.007	0.727
Vegetable intake_(log)_ on LDN (overweight/obesity)	−0.012	−0.034 to 0.009	0.266	−0.012	− 0.034 to 0.009	0.262
Indirect effect *100	0.003	−0.007 to 0.014	0.525	0.002	−0.009 to 0.012	0.737
Total effect *100	**0.793**	**0.346 to 1.239**	**0.001**	**0.873**	**0.414 to 1.332**	**0.000**
Model fit	–	AIC 6595.252	BIC_adj_ 6628.527	–	AIC 6176.544	BIC_adj_ 6243.095
Sugary drink intake (0 v > 0)						
ΔHbA_1c_ on LDN (overweight/obesity)	**0.008**	**0.003 to 0.012**	**0.001**	**0.009**	**0.004 to 0.013**	**0.000**
ΔHbA_1c_ on sugary drink intake	0.001	−0.006 to 0.008	0.731	−0.001	−0.008 to 0.006	0.857
Sugary drink intake on LDN (overweight/obesity)	**0.028**	**0.004 to 0.052**	**0.025**	**0.028**	**0.004 to 0.053**	**0.023**
Indirect effect *100	0.003	−0.015 to 0.021	0.725	−0.002	−0.022 to 0.019	0.860
Total effect *100	**0.791**	**0.346 to 1.237**	**0.000**	**0.871**	**0.414 to 1.329**	**0.000**
Model fit	–	AIC 7118.570	BIC_adj_ 7151.845	–	AIC 6715.416	BIC_adj_ 6781.966
**LDN: Insufficient fruit intake ** ***N*** ** = 1966**	**Unadjusted models**			**Adjusted models** ^a^		
Fruit serves (log transformed)	Estimate	95% CI	*P* value	Estimate	95% CI	*P* value
ΔHbA_1c_ on LDN (insufficient fruit intake)	**0.006**	**0.002 to 0.011**	**0.005**	**0.007**	**0.002 to 0.011**	**0.005**
ΔHbA_1c_ on fruit intake_(log)_	−0.004	− 0.011 to 0.002	0.145	− 0.004	− 0.011 to 0.002	0.188
Fruit intake_(log)_on LDN (insufficient fruit intake)	**−0.029**	**− 0.052 to − 0.007**	**0.010**	**−0.030**	**− 0.052 to − 0.007**	**0.010**
Indirect effect *100	0.013	−0.008 to 0.034	0.218	0.013	−0.009 to 0.035	0.246
Total effect *100	**0.650**	**0.203 to 1.097**	**0.004**	**0.668**	**0.214 to 1.123**	**0.004**
Model fit	–	AIC 6703.708	BIC_adj_ 6737.402	–	AIC 6258.888	BIC_adj_ 6326.276
Vegetable serves (log transformed)						
ΔHbA_1c_ on LDN (insufficient fruit intake)	**0.006**	**0.002 to 0.011**	**0.005**	**0.007**	**0.002 to 0.011**	**0.005**
ΔHbA_1c_ on vegetable intake_(log)_	−0.005	− 0.012 to 0.003	0.228	− 0.003	− 0.012 to 0.005	0.392
Vegetable intake_(log)_ on LDN (insufficient fruit intake)	−0.006	− 0.034 to 0.021	0.658	− 0.006	− 0.034 to 0.021	0.652
Indirect effect *100	0.003	−0.0110to 0.015	0.654	0.002	−0.008 to 0.012	0.659
Total effect *100	**0.650**	**0.201 to 1.098**	**0.005**	**0.664**	**0.209 to 1.119**	**0.004**
Model fit	–	AIC 6836.997	BIC_adj_ 6870.692	–	AIC 6395.732	BIC_adj_ 6463.120
Sugary drink intake (0 v > 0)						
ΔHbA_1c_ on LDN (insufficient fruit intake)	**0.006**	**0.002 to 0.0101**	**0.005**	**0.007**	**0.002 to 0.011**	**0.004**
ΔHbA_1c_ on sugary drink intake	0.002	−0.004 to 0.009	0.473	0.001	−0.006 to 0.008	0.861
Sugary drink intake on LDN (insufficient fruit intake)	**0.025**	**0.002 to 0.047**	**0.033**	**0.025**	**0.002 to 0.048**	**0.032**
Indirect effect *100	0.006	−0.011 to 0.022	0.481	0.002	−0.016 to 0.019	0.860
Total effect *100	**0.647**	**0.199 to 1.096**	**0.005**	**0.658**	**0.201 to 1.114**	**0.005**
Model fit	–	AIC 7365.307	BIC_adj_ 7399.001	–	AIC 6940.353	BIC_adj_ 7007.741

Numbers in bold are all statistically significant with p values

^a^Adjusted for area-level income and individual-level age, sex, employment status, education, marital status, and smoking status; Abbreviations: *AIC* Akaiki’s Information Criterion, *BIC*_*adj*_ sample size adjusted Bayesian Information Criterion, *CI* confidence interval, *HbA*_*1c*_ glycosylated haemoglobin, *LDN* local descriptive norm

**Table 4:** Results of structural equation models with change in BMI as outcome

**LDN: Overweight/obesity ** ***N*** ** = 1630**	**Unadjusted models**			**Adjusted models** ^a^		
Fruit serves (log transformed)	Estimate	95% CI	*P* value	Estimate	95% CI	*P* value
ΔBMI on LDN (overweight/obesity)	**0.029**	**0.014 to 0.045**	**0.000**	**0.020**	**0.004 to 0.036**	**0.012**
ΔBMI on fruit intake_(log)_	**−0.077**	**− 0.125 to − 0.029**	**0.002**	−0.029	− 0.074 to 0.015	0.198
Fruit intake_(log)_on LDN (overweight/obesity)	−0.017	−0.039 to 0.005	0.125	−0.018	− 0.040 to 0.004	0.114
Indirect effect *100	0.134	−0.043 to 0.311	0.139	0.052	−0.070 to 0.145	0.274
Total effect *100	**3.060**	**1.507 to 4.613**	**0.000**	**2.060**	**0.503 to 3.616**	**0.010**
Model fit	–	AIC 19,825.589	BIC_adj_ 19,856.662	–	AIC 19,569.671	BIC adj 19,631.817
Vegetable serves (log transformed)						
ΔBMI on LDN (overweight/obesity)	**0.030**	**0.015 to 0.046**	**0.000**	**0.020**	**0.004 to 0.036**	**0.012**
ΔBMI on vegetable intake_(log)_	**−0.054**	**−0.098 to − 0.009**	**0.017**	−0.023	−0.064 to 0.019	0.290
Vegetable intake_(log)_on LDN (overweight/obesity)	−0.004	−0.025 to 0.018	0.734	−0.004	− 0.026 to 0.017	0.714
Indirect effect *100	0.020	−0.093 to 0.134	0.728	0.009	−0.039 to 0.057	0.710
Total effect *100	**3.058**	**1.500 to 4.617**	**0.000**	**2.019**	**0.436 to 3.601**	**0.012**
Model fit	–	AIC 19,820.205	BIC_adj_ 19,851.278	–	AIC 19,557.197	BIC adj 19,619.343
Sugary Drink Intake (0 v > 0)						
ΔBMI on LDN (overweight/obesity)	**0.028**	**0.012 to 0.043**	**0.000**	**0.019**	**0.004 to 0.034**	**0.015**
ΔBMI on sugary drink intake	**0.088**	**0.055 to 0.122**	**0.000**	**0.044**	**0.011 to 0.077**	**0.009**
Sugary drink intake on LDN (overweight/obesity)	**0.030**	**0.003 to 0.057**	**0.030**	**0.030**	**0.003 to 0.058**	**0.028**
Indirect effect *100	0.266	−0.011 to 0.544	0.060	0.134	−0.035 to 0.303	0.119
Total effect *100	**3.054**	**1.503 to 4.604**	**0.000**	**2.054**	**0.486 to 3.622**	**0.010**
Model fit	–	AIC 20,356.077	BIC_adj_ 20,387.150	–	AIC 20,105.682	BIC adj 20,167.828
**LDN: Insufficient fruit intake N = 1673**	**Unadjusted models**			**Adjusted models** ^a^		
Fruit serves (log transformed)	Estimate	95% CI	*P* value	Estimate	95% CI	*P* value
ΔBMI on LDN (insufficient fruit intake)	0.010	− 0.003 to 0.023	0.118	0.001	−0.009 to 0.017	0.546
ΔBMI on Fruit intake_(log)_	**−0.080**	**−0.127 to − 0.033**	**0.001**	−0.032	− 0.076 to 0.013	0.161
Fruit intake_(log)_ on LDN (insufficient fruit intake)	**−0.026**	**−0.048 to − 0.005**	**0.015**	**−0.027**	**− 0.048 to − 0.006**	**0.012**
Indirect effect *100	**0.213**	**0.024 to 0.401**	**0.027**	0.086	−0.039 to 0.212	0.178
Total effect *100	1.236	−0.084 to 2.556	0.066	0.485	−0.826 to 1.796	0.469
Model fit	–	AIC 20,345.591	BIC_adj_ 20,377.028	–	AIC 20,083.362	BIC adj 20,146.237
Vegetable serves (log transformed)						
ΔBMI on LDN (insufficient fruit intake)	0.012	−0.001 to 0.026	0.079	0.005	−0.009 to 0.018	0.502
ΔBMI on vegetable intake_(log)_	**−0.059**	**−0.098 to − 0.013**	**0.007**	−0.026	− 0.066 to 0.015	0.216
Vegetable intake_(log)_on LDN (insufficient fruit intake)	−0.005	−0.028 to 0.020	0.693	−0.006	− 0.030 to 0.019	0.662
Indirect effect *100	0.029	−0.111 to 0.153	0.767	0.014	−0.048 to 0.077	0.910
Total effect *100	1.242	−0.039 to 2.623	0.067	0.468	−0.846 to 1.783	0.485
Model fit	–	AIC 20,352.891	BIC_adj_ 20,384.328	–	AIC 20,084.015	BIC adj 20,146.889
Sugary Drink Intake (0 v > 0)						
ΔBMI on LDN (insufficient fruit intake)	0.011	−0.002 to 0.024	0.098	0.004	−0.009 to 0.017	0.503
ΔBMI on sugary drink intake	**0.087**	**0.054 to 0.121**	**0.000**	**0.041**	**0.008 to 0.075**	**0.016**
Sugary drink intake on LDN (insufficient fruit intake)	0.017	−0.007 to 0.041	0.157	0.018	−0.006 to 0.042	0.141
Indirect effect *100	0.150	−0.065 to 0.365	0.172	0.074	−0.424 to 0.191	0.211
Total effect *100	1.270	−0.054 to 2.594	0.060	0.517	−0.790 to 1.823	0.438
Model fit	–	AIC 20,903.070	BIC_adj_ 20,934.507	–	AIC 20,644.999	BIC adj 20,707.873

Numbers in bold are all statistically significant with p values

^a^Adjusted area-level income and individual-level age, sex, employment status, education, marital status, and smoking status; Abbreviations: *AIC* Akaiki’s Information Criterion, *BIC*_*adj*_ sample size adjusted Bayesian Information Criterion, *BMI* body mass index, *CI* confidence interval, *LDN* local descriptive norm

Results from adjusted models are reported unless noted otherwise. Greater local descriptive norms for overweight/obesity were statistically significantly associated with fruit intake (inverse, β = − 0.025, 95% CI -0.047 to − 0.002, *p* = 0.030), sugary drink intake (β = 0.028, 95% CI 0.004 to 0.053, *p* = 0.023), and increasing HbA_1c_ over time (i.e., worsening, β = 0.009, 95% CI 0.004 to 0.013, *p* < 0.0001). Greater local descriptive norms for overweight/obesity were not associated with individual-level vegetable intake. Similarly, greater local descriptive norms for insufficient fruit intake were statistically significantly associated with individual-level fruit intake (inverse, β = − 0.030, 95% CI -0.052 to − 0.007, *p* = 0.010), sugary drink intake (β = 0.025, 95% CI 0.002 to 0.048, *p* = 0.032), and increasing HbA_1c_ (β = 0.007 CI 0.002 to 0.011, *p* = 0.005), but were not associated with vegetable intake. Fruit, vegetable and sugary drink intakes were not associated with change in HbA_1c_ and there were no statistically significant indirect effects between the local descriptive norms and change in HbA_1c_ through dietary behaviour.

In models assessing change in BMI, local descriptive norms for overweight/obesity were statistically significantly associated with individual-level sugary drink intake (β = 0.030, 95% CI 0.003 to 0.058, *p* = 0.028) and increasing BMI over time (β = 0.020, CI 0.004 to 0.036, *p* = 0.012), but not fruit or vegetable intake. Local descriptive norms for insufficient fruit intake were inversely associated with individual-level fruit intake (β − 0.027, 95% CI -0.048 to − 0.006, *p* = 0.012) but not statistically significantly associated with vegetable intake, sugary drink intake, or change in BMI. In unadjusted models, fruit, vegetable and sugary drink intakes were each associated with change in BMI, though only the estimate for sugary drink intake remained statistically significant after adjusting for covariates (sugary drink predicting change in BMI in local descriptive overweight/obesity norms models: β = 0.044, 95% CI 0.011 to 0.077, *p* = 0.009; and in local descriptive insufficient fruit intake norms models: β = 0.041, 95% CI 0.008 to 0.075, *p* = 0.016).

In unadjusted models, there was a statistically significant indirect effect between greater local descriptive norms for insufficient fruit intake and increasing BMI through individual-level fruit intake. However, this indirect effect was not statistically significant after accounting for covariates. There were no statistically significant indirect effects for either of the local descriptive norms and change in BMI in covariate-adjusted models.

## Discussion

This study found that local descriptive overweight/obesity norms were associated with individual-level fruit intake (inversely), sugary drink intake (positively), and worsening HbA_1c_ and BMI over 10 years. Similarly, local descriptive insufficient fruit intake norms were associated with individual-level fruit intake (inversely), sugary drink intake (positively), and worsening HbA_1c_ but not change in BMI. Unexpectedly, individual-level fruit, vegetable and sugary drink intakes were not associated with change in HbA_1c_. Fruit and vegetable intakes were associated with change in BMI (protective effect), but in unadjusted models only. Sugary drink intake alone was associated with change in BMI (worsening) in models accounting for individual-level covariates and area-level SES. There was a statistically significant indirect effect between greater local descriptive insufficient fruit intake norms and increase in BMI through lesser individual-level fruit intake, but again, this was only evident in models unadjusted for covariates.

Previous work by us found that greater local descriptive norms for overweight/obesity and insufficient fruit intake were each associated with worsening HbA_1c_ over 10 years [28]. These associations remained after accounting for the availability of fast food and healthful food resources and area-level SES. The current study builds on and expands this previous work by assessing associations with dietary intakes and change in BMI, and explicitly testing the potential mediating pathway of individual-level dietary intake.

As expected, greater local descriptive overweight/obesity norms were associated with increasing BMI, consistent with the few previous studies conducted. A Dutch study reported that normal weight adults residing in neighbourhoods with a greater prevalence of overweight were more likely to become overweight during 13 years of follow-up [39]. Similarly, analyses conducted within social networks have reported cross-sectional clustering and longitudinal spread (contagion) of obesity [35, 70]. The implications are that both subjective (i.e., within social networks) and local descriptive overweight/obesity norms affect individual-level health, and that further research is needed to elucidate any potential interactions between these sets of influences.

Little evidence exists to explain how descriptive norms influence individual body size and cardiometabolic risk. The clustering and spread of obesity within social networks is hypothesised to reflect socially shared norms about the acceptability of a larger body size, and socially shared behaviours [35]. If normative pressure to conform to healthy body weight is reduced where the descriptive norm denotes a larger, less healthy body size, this then could result in reduced motivation to follow diet and exercise health recommendations. Our findings support this notion. In addition, our results linking local insufficient fruit intake norms and individual-level fruit intake support the premise of socially shared behaviours at a local area level, thus both proposed mechanisms have support.

Previous studies of the influence of descriptive dietary norms on individual-level diet have observed associations like ours. A cross-sectional Australian study of disadvantaged women found that self-reported (and self-interpreted) perceived descriptive norms (defined neither in relation to one’s place of residence, nor in relation to a social group) were associated with self-reported dietary intakes [71]. Greater perceived norms for fast-food consumption and sugary drink intake were positively associated with greater fast food and sugary drink intakes, respectively. Similarly, greater perceived norms for healthy eating were positively associated with individual fruit and vegetable intake [71]. However, a cross-sectional design precluded inference on directionality, and self-reported information from the same individuals was used for the perceived norm *and* individual behaviour. Thus the findings could reflect a “false consensus effect” where beliefs about the behaviours of others are based on one’s own behaviours [72].

An experimental study within a university food court setting at lunch time, reported healthy descriptive norms messaging (“Every day more than 150 [name of university] students have a tossed salad for lunch here”) resulted in a greater number of individuals choosing to consume healthy food (defined as tossed salad) compared to a control condition [73]. For our study, that the local descriptive norms were associated with fruit and sugary drink intake but not vegetable intake may reflect greater difficulties in obtaining and regularly eating vegetables and a greater dislike of vegetables compared with fruits and sugary drinks. Research on differences in perceptions around fruits, vegetables and sugary drinks and influences that may moderate normative effects on dietary intakes is needed.

This study did not find associations between individual-level fruit, vegetable, and sugary drink intakes and change in HbA_1c_. Further, even though we observed associations between dietary intakes and change in BMI, only the association between sugary drink intake and increasing BMI remained after accounting for sociodemographic covariates and area-level SES. These findings were unexpected, as a greater consumption of fruits and vegetables, and a restricted intake of sugary drink are widely-accepted predictors of healthy body weight and protective against cardiometabolic disease [4, 10]. Not all studies concur, however. A cross-sectional study of Malaysian adults with type 2 diabetes reported a greater intake of vegetables was associated with greater HbA_1c_ level, though this contrary finding could be due to the cooking methods used as vegetables are often stir-fried or cooked with water or coconut milk in Malaysia [74]. Previous studies (e.g., [15–17, 75]) have reported greater fruit and vegetable intakes amongst individuals who were female, older, and with higher incomes, factors which were included as covariates in analytic models for the current study. Consequently, the loss of association for fruit and vegetable intake with change in BMI upon model adjustment may be due to over-adjustment. The lack of association between dietary intakes and HbA_1c_, however, is not explained by covariance with other measures as this lack of association was apparent in unadjusted and adjusted models. This may reflect imprecision in the measurement of self-reported diet behaviour which could bias results to the null.

The only indirect effect found was in unadjusted models and only between local descriptive insufficient fruit intake norms and change in BMI through individual fruit intake. There were no statistically significant indirect effects of local descriptive norms on change in HbA_1c_ or BMI through dietary behaviours in adjusted models. This reflects the lack of associations between fruit and vegetable intakes and change in BMI and HbA_1c_. The lack of a statistically significant indirect effect for sugary drink intake as the mediator between local descriptive overweight/obesity norms and change in BMI may be due to the size of the coefficients for the associations on the pathway. In unadjusted models this indirect effect was close to statistical significance. Though none of the indirect effects were significant in this sample, this may not reflect a broader lack of association, particularly as the findings were potentially driven by the unexpected lack of association between dietary intake and health outcomes. Future research testing individual-level dietary intake as a link between local descriptive health-related norms and health outcomes, with different samples, would help unravel whether this issue is specific to our sample.

Assessment of other potential mechanisms linking local descriptive norms to health outcomes is also needed. For associations between local descriptive overweight/obesity norms and HbA_1c_ or BMI, this could include physical activity behaviour as physical activity is implicated in each health outcome and may be motivated by local normative pressures to conform. However, our analysis assessing the indirect effect of local descriptive overweight/obesity norms on change in HbA_1c_ through physical activity behaviour found only a small partial mediation effect [76]. Other potential pathways include other behavioural factors (e.g., other diet factors, sedentary behaviour) as well as psychosocial and stress-related pathways [77]. Moreover, the effect of such pathways may be modified by other individual or environmental factors [28, 78].

Significant associations between local descriptive norms, dietary intakes and health outcomes reported in this study highlight the importance of descriptive norms to health outcomes. Interventions aiming to improve dietary behaviour and health outcomes must consider the local normative environment. Areas with adverse local normative conditions may be resistant to behaviour change interventions. Though injunctive norms messages can promote the importance of healthful behaviour, injunctive norms can be undermined by opposing descriptive norms [22, 79, 80]. Importantly, descriptive norms may be used to promote healthful behaviours. This could include descriptive norms messaging strategies aligned with injunctive norms as well as environmental strategies aiming to influence perceptions of normative behaviour.

Individuals do not wish to deviate substantially from descriptive norms, therefore, the framing of descriptive norms messages needs to be carefully considered [81]. Messages need to be positively framed with a majority ruling (e.g., most people enjoy eating fruit regularly) [78, 82]. Environmental strategies can be used to manipulate perceptions of norms, thus hopefully positively influencing behaviour. One study manipulated the size of shopping cart partitions identified as being for fresh fruits and vegetables, reporting larger partitions increased purchasing of these items [83]. Other studies have reported a portion size effect where a larger portion size is associated with greater food intake (e.g., [84]). Policies could be used to influence such factors, along with food pricing, availability of healthful and unhealthful foods, and product placement (e.g., within supermarkets), all of which may influence food purchasing and intake, and the social acceptability of food options.

### Strengths and limitations

Strengths of this study include the use of an explicit definition of the type of norm being assessed, and the construction of local descriptive norms from a wholly separate survey, thus avoiding same sample bias and the false consensus effect [72, 85]. While both samples (NWAHS and SAMSS) used in this research were drawn from the same population and are each considered broadly reflective of that population, there remains the possibility of some level of sampling bias which could vary between samples. However, in representing environmental exposures that cannot be constructed from locational databases, the use of a separate survey sample is recommended and has commonly been used in research regarding place influences on health (e.g., [86–88].

Our longitudinal design supports causal inference through temporality of observations, and reduces the likelihood of reverse causation [40]. Most studies reporting associations between where we live and health behaviours or other outcomes are cross-sectional. Moreover, as much of the research reporting the influence of food behaviour related norms has been in experimental settings, and based on individuals, assessing the relationships within a free-living population adds external validity to such findings. The NWAHS cohort is broadly representative of the Adelaide population and the findings of this study should be generalisable to similar populations residing within similar urban residential environments.

The outcome measures used in this study, HbA_1c_ and BMI, were clinically assessed and each expressed in a continuous format. The use of clinically assessed measures avoids self-report bias, particularly relevant to BMI. Expressing the outcome measure in a continuous format provides greater information on severity of risk, more precisely reflecting the magnitude of change over time. Such measures provide greater statistical power than categorised measures [89]. Individual-level dietary and sociodemographic information was self-reported and is thus subject to potential self-report bias. Similarly, local descriptive norms were constructed from aggregated self-reported information. If BMI is under-reported and fruit intake over-reported [90, 91], then the absolute values of the local descriptive norms (i.e., buffer specific prevalence rates) may be under-estimated. However, the *relative* comparison of local descriptive norms should not be greatly affected.

Finally, though this study sought to minimise confounding and reduce bias due to cohort attrition by including covariates within fitted models, there remains the possibility of residual confounding due to unmeasured influences. Of note, changes in local descriptive norms may have co-occurred with changes in individual-level behaviour in response to some unmeasured factor. Environmental influences on individual-level health outcomes may act through multiple interdependent pathways whilst being subject to an assortment of potential confounders. Complex systems approaches, such as agent-based modelling, may be useful in efforts seeking to understand the complex causal processed likely to be at work [92–95].

## Conclusion

Local descriptive norms for overweight/obesity and insufficient fruit intake were associated with less healthful dietary intakes of fruits, vegetables and sugary drinks, and worsening trajectories of cardiometabolic risk (HbA_1c_ and BMI). Local descriptive health-related norms are arguably under-recognised influences on individual-level behaviour and outcomes. Local descriptive norms therefore require consideration in intervention strategies aiming to reduce population risk of chronic diseases.

## Abbreviations

AIC: Akaiki’s Information Criterion
BIC_adj_: sample size adjusted Bayesian Information Criterion
BMI: Body mass index
CATI: Computer-Assisted Telephone Interview
CDs: Census Collection Districts
CI: Confidence interval
CVD: Cardiovascular disease
FIML: Full information maximum likelihood
GIS: Geographic information system
HbA_1c_: Glycosylated haemoglobin
ICC: Intraclass correlation
LDN: Local descriptive norms
NWAHS: North West Adelaide Health Study
OECD: Organisation for Economic Co-operation and Development
PAMS: Place and Metabolic Syndrome
SAMSS: South Australian Monitoring and Surveillance System
SD: Standard deviation
SEM: Structural equation modelling
SES: Socioeconomic status
W1: Wave 1
W2: Wave 2
